# Diagnostic Performance of Tuberculosis-Specific IgG Antibody Profiles in Patients with Presumptive Tuberculosis from Two Continents

**DOI:** 10.1093/cid/cix023

**Published:** 2017-01-25

**Authors:** Tobias Broger, Robindra Basu Roy, Angela Filomena, Charles H. Greef, Stefanie Rimmele, Joshua Havumaki, David Danks, Nicole Schneiderhan-Marra, Christen M. Gray, Mahavir Singh, Ida Rosenkrands, Peter Andersen, Gregory M. Husar, Thomas O. Joos, Maria L. Gennaro, Michael J. Lochhead, Claudia M. Denkinger, Mark D. Perkins

**Affiliations:** 1 FIND, Geneva, Switzerland; 2 Section of Paediatrics, Imperial College London, United Kingdom; 3 Natural and Medical Sciences Institute at the University of Tübingen, Reutlingen, Germany; 4 MBio Diagnostics Inc., Boulder, Colorado; 5 Department of Philosophy, Carnegie Mellon University, Pittsburgh, Pennsylvania; 6 Lionex GmbH, Braunschweig, Germany; 7 Statens Serum Institut, Copenhagen, Denmark; 8 Public Health Research Institute, Rutgers New Jersey Medical School, New Brunswick

**Keywords:** serologic tests, tuberculosis, biomarkers, antibodies, diagnosis

## Abstract

**Background.:**

Development of rapid diagnostic tests for tuberculosis is a global priority. A whole proteome screen identified *Mycobacterium tuberculosis* antigens associated with serological responses in tuberculosis patients. We used World Health Organization (WHO) target product profile (TPP) criteria for a detection test and triage test to evaluate these antigens.

**Methods.:**

Consecutive patients presenting to microscopy centers and district hospitals in Peru and to outpatient clinics at a tuberculosis reference center in Vietnam were recruited. We tested blood samples from 755 HIV–uninfected adults with presumptive pulmonary tuberculosis to measure IgG antibody responses to 57 *M. tuberculosis* antigens using a field-based multiplexed serological assay and a 132-antigen bead-based reference assay. We evaluated single antigen performance and models of all possible 3-antigen combinations and multiantigen combinations.

**Results.:**

Three-antigen and multiantigen models performed similarly and were superior to single antigens. With specificity set at 90% for a detection test, the best sensitivity of a 3-antigen model was 35% (95% confidence interval [CI], 31–40). With sensitivity set at 85% for a triage test, the specificity of the best 3-antigen model was 34% (95% CI, 29–40). The reference assay also did not meet study targets. Antigen performance differed significantly between the study sites for 7/22 of the best-performing antigens.

**Conclusions.:**

Although *M. tuberculosis* antigens were recognized by the IgG response during tuberculosis, no single antigen or multiantigen set performance approached WHO TPP criteria for clinical utility among HIV-uninfected adults with presumed tuberculosis in high-volume, urban settings in tuberculosis-endemic countries.

## Summary

Samples from >750 patients with presumptive TB in two continents were tested with a field-based IgG serological assay incorporating 57 *M. tuberculosis* antigens, and showed that responses to single antigens or multiple antigen sets have no clinically useful discriminating power.

Despite advances in rapid molecular diagnostic techniques for tuberculosis, an unmet need remains for a point-of-care nonsputum-based test [1]. The World Health Organization (WHO) has defined high-priority target product profiles (TPPs) for tuberculosis diagnostics [2]. These include a rapid nonsputum-based test for detecting tuberculosis with the purpose of initiating tuberculosis treatment on the same day (tuberculosis detection test henceforth) and a community-based triage or referral test for differentiating patients with signs and symptoms of active tuberculosis who need referral for further confirmatory testing from those who do not have tuberculosis (tuberculosis triage test henceforth). Serological tests such as lateral flow assays are appealing for these applications due to their simplicity, lack of specimen processing requirements, and implementation record. However, performance of serodiagnostics for tuberculosis has been disappointing, leading the WHO to issue a strong recommendation against the use of serological tests commercialized for the diagnosis of active tuberculosis [3, 4]. In the WHO policy statement, further research was encouraged, specifically with representative populations with presumptive tuberculosis in studies with prospective follow-up and blinding [4].

One reason why serological diagnosis of tuberculosis has been challenging is that immunoglobulin G (IgG) responses to tuberculosis are heterogeneous across patient populations and most tuberculosis serologic tests use a single or small number of antigens [5–9]. Therefore, FIND, a nonprofit organization that enables diagnostic development for poverty-related diseases, has been systematically working to define the diagnostic potential of tuberculosis serological responses. Using a high-throughput expression system, the entire tuberculosis proteome was tested with globally collected sera from more than 500 patients with tuberculosis disease or nontuberculous pulmonary disease [6]. Although antibody responses were detected against approximately 10% (or 484 proteins) of the bacterial proteome, a much smaller fraction generated responses that might distinguish active tuberculosis from nontuberculous pulmonary disease. We prioritized proteins that exhibited the best discriminating capacity toward the development of new tuberculosis detection or tuberculosis triage tests.

Here, we report the diagnostic performance of a custom, portable multiplexed serology assay (MBio Diagnostics) that was used to measure antibody responses to 57 selected antigens in blood from more than 750 prospectively enrolled adults with presumptive pulmonary tuberculosis at 2 trial sites in Vietnam and Peru. Antibody response to a broader set of 132 antigens in frozen sera was also measured using a multiplex bead-based Luminex assay at a reference research laboratory.

## METHODS

### Study Design

**Figure 1.**
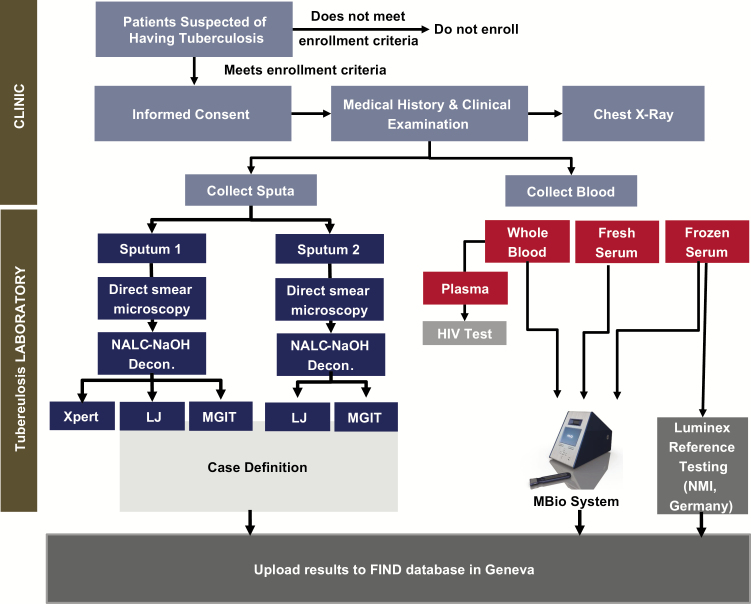
Patient and specimen flow. Abbreviations: HIV, human immunodeficiency virus; LJ, Löwenstein–Jensen solid medium; MGIT, BACTEC mycobacteria growth indicator tube 960 culture; NALC-NaOH Decon., N-acetyl-L-cysteine-sodium hydroxide decontamination; NMI, Natural and Medical Sciences Institute at the University of Tübingen; Xpert, Xpert® MTB/RIF.

Adults who presented to ambulatory healthcare centers with symptoms suggestive of pulmonary tuberculosis in Peru and Vietnam were consecutively enrolled in the study. The site in Peru is a research institute in Lima that recruits patients from microscopy centers and district hospitals in a high tuberculosis prevalence area. The site in Vietnam is a tuberculosis and lung disease reference center in Ho Chi Minh City that recruits patients from its outpatient clinic. Inclusion and exclusion criteria for entry into the trial are detailed in Supplementary Table 1, and criteria to exclude recruited patients from the analysis are provided in Supplementary Appendix 1. Patient characteristics, comorbidities, and tuberculosis history were recorded. Human immunodeficiency virus (HIV)–positive individuals were excluded from this feasibility study in view of potentially altered serological responses [10]. Written informed consent was obtained from all patients. Study participation did not alter the standard of care. Figure 1 depicts the patient and specimen flow. Recruitment was done in 2 phases: phase 1 was used to validate the field-based MBio platform against the reference assay before proceeding to the larger phase 2.

### Sample Procedures

At least 2 sputum samples were collected from each patient and tested with direct smear microscopy, solid and liquid culture, and with Xpert® MTB/RIF (Cepheid, Sunnyvale), when available. The presence of *Mycobacterium tuberculosis* complex in cultures was confirmed by MPT 64 antigen detection (Capilia TB, Tauns Laboratories, Japan). Serum (SE), whole blood (WB), and frozen serum (FZ) were collected for analysis (Supplementary Appendix 2).

### Case Definitions

Supplementary Table 2 summarizes the case definitions. Patients were categorized based on clinical and microbiological results. Patients with positive *M. tuberculosis* cultures were diagnosed as definite tuberculosis and subcategorized into smear-positive and smear-negative groups. Participants who were smear negative and culture negative but responded to empiric tuberculosis treatment were classified as “clinical tuberculosis.” Participants who were smear negative and culture negative on all sputum samples and who exhibited symptom resolution in the absence of tuberculosis treatment at the 2-month follow-up visit were classified as “non-tuberculosis disease.”

### Antigen Selection

**Figure 2.**
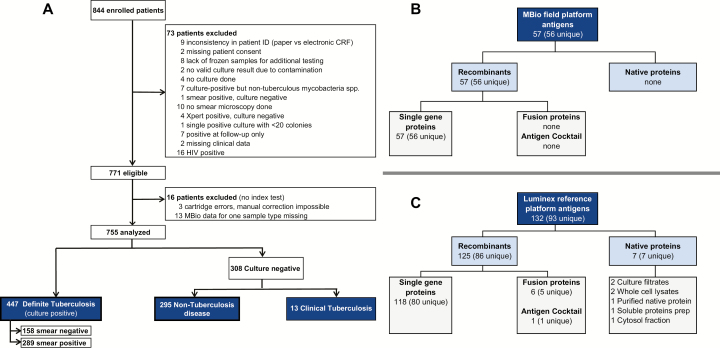
Summary of patient and antigen selection. *A,* Recruitment flow diagram for patients enrolled in the study. *B,* MBio field platform antigens. *C,* Luminex reference platform antigens. All MBio field platform antigens were also measured on the Luminex reference platform. Some of the antigens used have the same Rv number (see http://tuberculist.epfl.ch/) and amino acid sequence, but were provided by different suppliers or expressed in different production strains. Therefore, the number of unique antigens is shown in brackets. A comprehensive list can be found in Supplementary Table 3. Abbreviations: CRF, Case report form; HIV, human immunodeficiency virus.

The antigen selection process is described in Supplementary Appendix 3 and Supplementary Figure 1. In summary, 62 antigens from the whole proteome screen [6] were selected for expression and purification on the basis of odds ratios, area under the curve (AUC), or high model importance in random forest analysis. An additional 41 antigens were selected in independent research studies performed by institutions that collaborate with FIND. The resulting antigen panel was prescreened with 1321 patient sera by a multiplex Luminex assay [11] to define the subset of 57 recombinant antigens (Figure 2B) used in this study. To validate the down-selection of target antigens, we retained the Luminex assay and added additional antigens such that this platform contained 132 antigens (including recombinant proteins, fusion proteins, antigen cocktails, and native protein preparations; Figure 2C). There were 93 unique antigens. The antigen list and expression, purification, and quality details are listed in Supplementary Appendix 4 and Supplementary Table 3.

### MBio Multiplexed Serological Assay

The MBio multiplexed immunoassay system uses disposable cartridges that have a 2-dimensional array of antigens and controls along with a laptop-connectable reader (SnapEsi; Supplementary Appendix 5) [12]. Briefly, diluted sample is added to the cartridge and, after an incubation and wash, fluorescently labeled anti-human IgG is used to measure antigen-bound human IgG for each spot in the array.

### Serological Luminex Assay

The multiplex bead-based Luminex assay, which measures IgG antibodies in FZ, was performed as described in Planatscher et al [13] and Supplementary Appendix 6.

### Diagnostic Performance of Single Antigens and Antigen Models

The WHO TPP for the tuberculosis detection test and tuberculosis triage test are summarized in Table 1, alongside the study targets for the MBio and Luminex platforms. The performance of antigens (or antigen sets) were evaluated against the following 3 targets: tuberculosis detection test sensitivity at preset specificity, tuberculosis triage test specificity at preset sensitivity, and AUC (Supplementary Appendix 8).

**Table 1.** World Health Organization–Endorsed Performance Targets and Prespecified Study Targets for Judging Performance of Antigen Panels for Diagnosis of Tuberculosis

WHO Target Product Profile Name	**WHO Target Product Profile Requirements**	**Study Target** **Luminex Reference Platform**	**Study Target** **MBio Field Platform Target**
Rapid, biomarker-based nonsputum-based test for detecting tuberculosis **Tuberculosis detection test**	Specificity ≥98% Sensitivity ≥65%	Specificity set at 95% Sensitivity target: ≥60% Frozen serum	Specificity set at 90% Sensitivity target: ≥55% Indeterminate rate >10%Fresh diluted serum or diluted whole blood”
	Target population: “adults and children including those who are HIV-positive and with presumptive active pulmonary TB or extra-pulmonary TB in countries with a medium prevalence to a high prevalence of TB as defined by WHO.”	Study population: Prospective consecutively presenting HIV-uninfected adults with presumptive pulmonary tuberculosis attending: • Microscopy centers or district hospitals in Lima, Peru (WHO high-prevalence country) • Outpatient clinic at tuberculosis and lung disease hospital, Ho Chi Minh City, Vietnam (WHO high-prevalence country)	
Community-based triage or referral test to identify people with presumptive tuberculosis **Tuberculosis triage test**	Sensitivity ≥90% Specificity ≥70%	Sensitivity set at 90% Specificity target: ≥60% Frozen serum	Sensitivity set at 85% Specificity target: ≥55% Indeterminate rate >10%Fresh diluted serum or diluted whole blood
	Target population: “adults and children with signs and symptoms of active pulmonary TB in countries with a medium prevalence to a high prevalence of TB as defined by WHO.”	Study population: Prospective consecutively presenting HIV-uninfected adults with presumptive pulmonary tuberculosis attending: • Microscopy centers or district hospitals in Lima, Peru (WHO high-prevalence country) • Outpatient clinic at tuberculosis and lung disease hospital, Ho Chi Minh City, Vietnam (WHO high-prevalence country)	

Abbreviations: HIV, human immunodeficiency virus; WHO, World Health Organization. Target Product Profiles can be accessed on-line under http://www.who.int/tb/publications/tpp_report/en/ [2]

### Statistical Analysis for Multiantigen Combinations

Generalized linear models (GLMs), generalized additive models (GAMs), and naive Bayes (NB) with 8-fold cross-validation were used to test all possible 3-antigen combinations and to identify a subset of antigen combinations by stepwise regression for each platform and sample type (Supplementary Appendixes 9 and 10). The models were set to optimize sensitivity at preset specificity for tuberculosis detection tests, specificity at preset sensitivity for the tuberculosis triage tests, and AUC.

## RESULTS

### Study Participants

A total of 844 eligible patients with presumptive tuberculosis were recruited to the study (Figure 2A). A total of 73 were excluded from analysis due to new diagnosis of HIV or discrepancies in study documentation, microbiological test results, or study sample collection (Figure 2A, Supplementary Appendix 1). There were 16 additional exclusions due to missing MBio data or cartridge errors (Figure 2A). A total of 755 adults were therefore eligible for analysis with 447 definite tuberculosis cases, 13 cases of clinical tuberculosis, and 295 of non-tuberculosis disease (Figure 2A, Table2).

**Table 2.** Characteristics of Study Participants

Characteristic	Peru			Vietnam			Total		
	Non- Tuberculosis Disease (n = 167)	Definite Tuberculosis (n = 230)	Clinical Tuberculosis (n = 8)	Non-Tuberculosis Disease (n = 128)	Definite Tuberculosis (n = 217)	Clinical Tuberculosis (n = 5)	Non- Tuberculosis Disease (n = 295)	Definite Tuberculosis (n = 447)	Clinical Tuberculosis (n=13)
Age, y									
	41.5 ± 15.7 (*P* < .0001)^a^	31.6 ± 14.0	29.0 ± 13.1	45.0 ± 16.3 (*P* < .0001)^a^	37.6 ± 13.5	56 ± 10.2	43.0 ± 16.0 (*P* < .0001)^a^	34.5 ± 14.1	29.3 ± 18.0
Gender									
Male	75 (44.9%) (*P* = 0.001)^b^	144 (62.6%)	4 (50.0%)	95 (74.2%) (*P* = 0.40)^b^	146 (67.2%)	4 (80.0%)	170 (57.6%) (*P* = 0.13)^b^	290 (64.9%)	8 (61.5%)
Female	92 (55.1%)	86 (37.4%)	4 (50.0%)	33 (25.8%)	71 (32.7%)	1 (20.0%)	125 (43.4%)	157 (35.1%)	5 (38.5%)
Enrollment body mass index									
Underweight (<18.5)	2 (1.2%) (*P* < .0001)^c^	21 (9.1%)	2 (25.0%)	46 (36.0%) (*P* = .0006)^c^	96 (44.2%)	2 (40.0%)	48 (16.3%) (*P* < .0001)^c^	117 (26.2%)	4 (30.8%)
Normal (≥18.5 + <25)	97 (58.1%)	163 (70.9%)	6 (75.0%)	75 (58.6%)	118 (54.4%)	3 (60.0%)	172 (58.3%)	281 (62.9%)	9 (69.2%)
Overweight (≥25 + <30)	50 (29.9%)	39 (17.0%)	0 (0%)	7 (5.5%)	3 (1.4%)	0 (0%)	57 (19.3%)	42 (9.4%)	0 (0%)
Obese (≥30)	16 (9.6%)	7 (3.0%)	0 (0%)	0 (0%)	0 (0%)	0 (0%)	16 (5.4%)	7 (1.6%)	0 (0%)
Tuberculosis history									
No	124 (74.3%) (*P* = .0004)^b^	203 (88.3%)	7 (87.5%)	24 (18.8%) (*P* < .0001)^b^	88 (40.6%)	5 (100%)	148 (50.2%) (*P* < .0001)^b^	291 (65.1%)	12 (92.3%)
Yes	40 (24.0%)	26 (11.3%)	0 (0%)	13 (10.2%)	8 (3.7%)	0 (0%)	53 (18.0%)	34 (7.6%)	0 (0%)
Not done	3 (1.8%)	1 (0.4%)	1 (12.5%)	91 (71.1%)	121 (55.8%)	0 (0%)	94 (31.9%)	122 (27.3%)	1 (7.7%)
Smear status^d^,^e^									
Smear negative	167 (100.0%)	50 (21.7%)	8 (100%)	128 (100%)	108 (49.8%)	5 (100%)	295 (100%)	158 (35.4%)	13 (100%)
Smear positive	0 (0%)	180 (78.3%)	0 (0%)	0 (0%)	109 (50.2%)	0 (0%)	0 (0%)	289 (64.7%)	0 (0%)
Xpert® MTB/RIF^e^									
Negative	120 (72%)	10 (4%)	5 (63%)	122 (95%)	46 (21%)	4 (80%)	242 (82%)	56 (12%)	9 (69%)
Positive	0 (0%)	167 (73%)	0 (0%)	0 (0%)	171 (79%)	0 (0%)	0 (0%)	338 (76%)	0
No result (indeterminate or no result)	47 (28%)	53 (23%)	3 (37%)	6 (5%)	0 (0%)	1 (20%)	53 (18%)	53 (12%)	4 (31%)

Data from phases 1 and 2, excluding patients whose final diagnosis was indeterminate.

a Logistic regression.

b Fisher exact test.

c Mantel-Hanzel χ^2^ test.

d No *P* value since it is used to calculate outcome, final diagnosis.

e Smear-positive/culture-neegative and Xpert® MTB/RIF -positive/culture-negative individuals were excluded from the analysis where smear or Xpert® MTB/RIF results were available.

### Validation of MBio and Luminex Platforms

In phase 1, samples from 184 patients (Supplementary Table 4) were used to validate the MBio system, which showed high concordance with the Luminex assay (Supplementary Appendix 7) and met all endpoints at both sites [14]. The Luminex assay had intra- and inter-assay coefficients of variation of <20% for 128/132 antigens (Supplementary Appendix 6). Antigen coupling efficiency of all His-tagged antigens (n = 125) was confirmed using an anti-His antibody (Supplementary Table 11). MBio analysis was based on all 755 patients; for Luminex, we focused on the larger phase 2 (571 valid results), as the signal ranges in the 2 experimental runs did not permit combination.

### Single Antigen Performance

**Figure 3.**
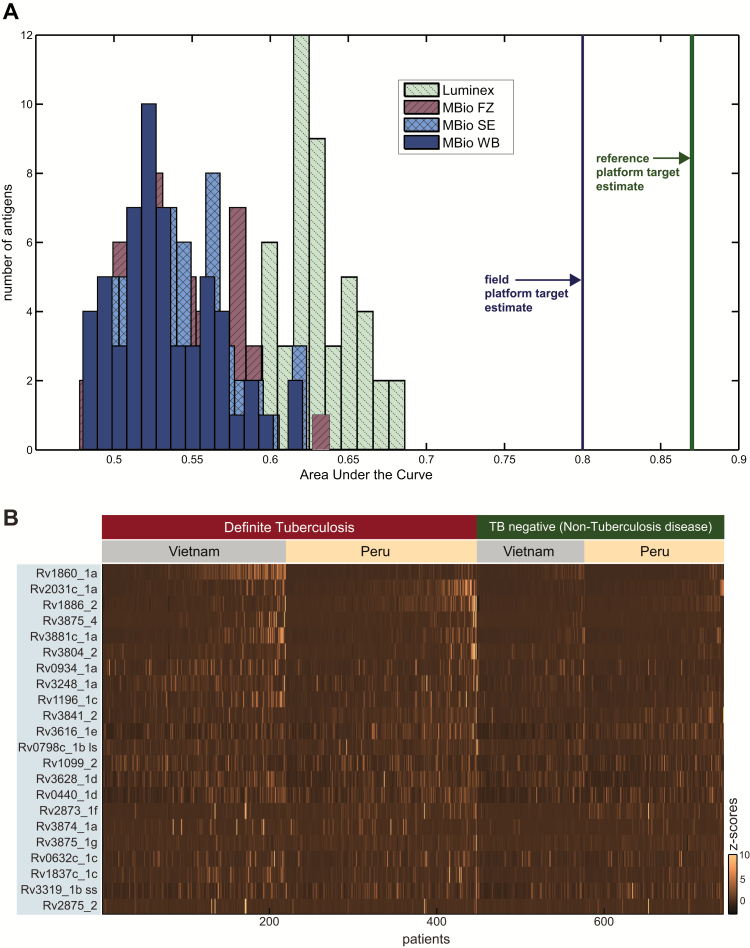
Summary of individual antigen performance. *A,* Area under the curve (AUC) histograms by sample type and platform for the 57 antigens that were measured on both platforms. AUCs of fresh (serum, whole blood) and frozen (serum) samples from MBio are in the same range, and no difference per sample type could be observed. There was a tendency for higher AUCs from Luminex compared to MBio, but differences were only significant for 5 antigens (Supplementary Table 5). Approximate AUC targets (blue and green lines) were estimated based on study targets (AUC estimated by receiver operating characteristic (ROC) curve fitting through the study target points using a binormal model) and were not met by single antigens. *B,* Heterogeneous antibody response pattern of 755 tuberculosis patients. The heat map shows the reactivity of sera (standardized *z*-scores of MBio fresh serum intensity signals) to the 22 highest ranked MBio antigens. Abbreviations: FZ, frozen serum; SE, fresh serum; WB, fresh whole blood.

When we analyzed performance of the 57 single antigens on the MBio platform, we identified 22 top-ranking antigens (Supplementary Table 5). The best antigen for a tuberculosis detection test with a predefined specificity of 90% was Rv2031c_1a, with highest sensitivity of 25% (95% confidence interval [CI], 21–29) on fresh serum. The best antigen for a tuberculosis triage test with a predefined sensitivity of 85% was Rv1860_1a, with highest specificity of 32% (95% CI, 26–37) on frozen serum (Supplementary Figure 2). Comparing the performance of the same 57 antigens on the Luminex platform, we found that although AUCs tended to be higher on Luminex, only 5 of the 22 top-ranked antigens performed significantly better than on MBio (Figure 3A, Supplementary Table 5). The highest AUC was 0.69 (Rv2031c_1a) on Luminex and 0.64 (RV1860_1a, MBio FZ) on MBio (Supplementary Table 5).

When we analyzed the performance of the 75 additional antigens that were only measured on the Luminex platform, we identified antigens that performed slightly better (Supplementary Table 6), with the sensitivity of the best antigen for the tuberculosis detection test being 31% (95% CI, 27–36) for Rv1886_5. The highest AUC of a single antigen on Luminex was 0.72 from WCL_H37Rv_6.

### Influence of Study Site and Previous History of Tuberculosis on Performance

Given the study design and the study participants’ characteristics (Table 2), we carried out post hoc subanalyses to determine whether performance varied between the study sites and by the participants who had a previous history of tuberculosis.

**Table 3A.** Number of Patients Grouped by Patient Classification for the Best 3-Antigen Model (Rv1860_1a, Rv1886_2, Rv3881c_1a, GLM, fresh serum, MBio) for a Tuberculosis Detection Test with Preset Specificity at 90%.

	**Definite** **Tuberculosis** **(n = 447)**	**Clinical** **Tuberculosis** **(n = 13)**	**Non**- **Tuberculosis** **Disease** **(n = 295)**	**Total** **(n = 755)**
**Predicted positive by the best 3-antigen** **model**	156	0	29	185
**Predicted negative by the** **best 3-antigen** **model**	291	13	266	570

Figure 3B shows immunological responses of 755 patients to the 22 highest ranked antigens on the MBio assay categorized by study site. Patient-to-patient antibody response was variable, and false-positive reactivity can be observed in the non-tuberculosis disease groups of both sites. Antibody responses to some antigens, including the top 3 MBio antigens, were dependent on study site. Antibody response against Rv1860_1a was higher in the Vietnam cohort, resulting in a significantly higher tuberculosis detection test sensitivity (38% in Vietnam vs 10% in Peru). Antibody response against Rv2031c_1a and Rv1886_2 had significantly higher AUCs in Peru due to increased reactivity in the Peruvian definite tuberculosis group (Figure 3B, Supplementary Table 9).

Given the relatively high proportion of patients with a history of previous tuberculosis in our cohort (Table 2), we also carried out a subanalysis where patients with a history of tuberculosis or unknown history of tuberculosis were excluded (Supplementary Table 10). There were no significant differences in AUC when compared to analysis of the whole cohort.

### Three-Antigen Models

We then determined whether combinations of responses to 3 antigens would improve diagnostic performance. We used GAM, GLM, and NB models for all possible 3-antigen sets for the 57 antigens measured on MBio (29 260 input sets). The best tuberculosis detection sensitivity was 35% (95% CI, 31–40) for the combination of Rv1860_1a, Rv1886_2, and Rv3881c_1a (Table 3A). The best tuberculosis triage test specificity was 34% (95% CI, 29–40) for the combination of Rv1860_1a, Rv1886_2, and Rv3874_1a (Table 3B), which is significantly above the performance of the best single antigens. The frequency of the top 22 ranked single antigens in the best 3-antigen models is presented in Supplementary Table 7, confirming that antigens with a high single-antigen performance occur very frequently in the best models.

**Table 3B.** Number of Patients Grouped by Patient Classification for the Best 3-Antigen Model (Rv1860_1a, Rv1886_2, Rv3874_1a, GAM, fresh serum, MBio) for a Tuberculosis Triage Test with Preset Sensitivity at 85%.

	**Definite** **Tuberculosis** **(n = 447)**	**Clinical** **Tuberculosis** **(n = 13)**	**Non**- **Tuberculosis** **Disease** **(n = 295)**	**Total** **(n = 755)**
**Predicted positive by the best 3-antigen** **model**	380	9	194	583
**Predicted negative by the** **best 3-antigen** **model**	67	4	101	172

Abbreviations: GAM, general additive model; GLM, generalized linear model.

### Multiantigen Models

We also looked at models that contained larger numbers of antigens, with stepwise regression yielding 4 to 12 antigens depending on model and sample type. The best model for tuberculosis detection involved 7 antigens and had a sensitivity of 36% (95% CI, 32–41). The best model for tuberculosis triage test had specificity of 37% (95% CI, 31–43) based on 9 antigens. We further conducted the same subanalyses using only the 439 patients with no tuberculosis history. Similar to the subanalysis for single antigens, performance did not significantly improve (tuberculosis detection test sensitivity of 37% and tuberculosis triage test specificity of 40% respectively).

### Evaluation of Diagnostic Performance Against WHO Target Product Profiles

**Figure 4.**
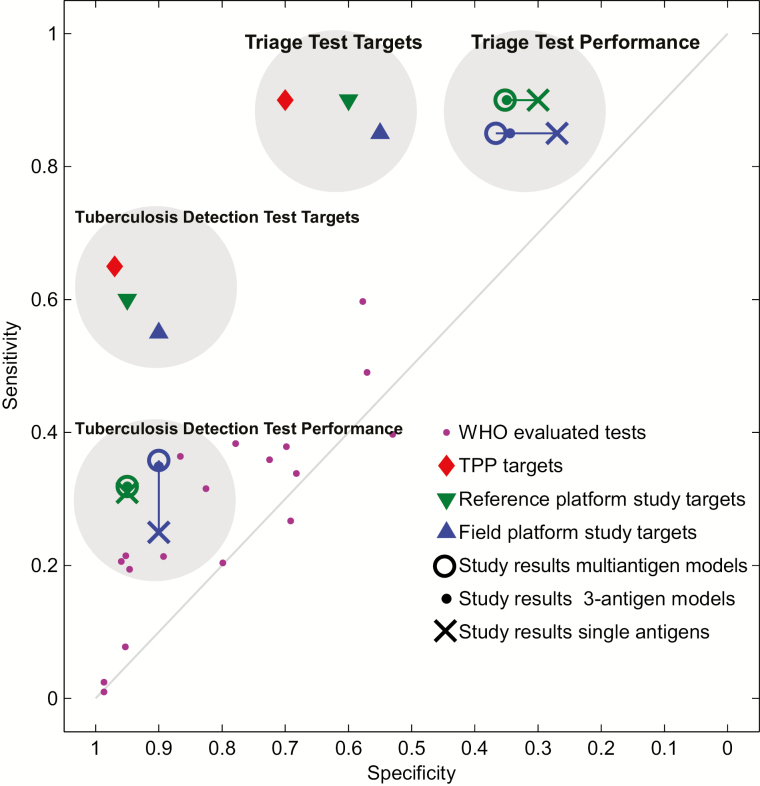
Study targets for a tuberculosis detection test and tuberculosis triage test (▲ for MBio, ▼ for Luminex) were not met by the best single antigen, the best 3-antigen model, or the best multiantigen model on both platforms (blue symbols for MBio and green symbols for Luminex) and are even further away from the needed performances as defined in 2 high-priority target product profiles (♦). Purple dots (•) depict the performance of commercial rapid diagnostic tests for tuberculosis that were evaluated by the World Health Organization in 2008. Abbreviations: TPP, target product profile; WHO, World Health Organization.

Finally, we compared the performance of single antigens, 3-antigen models, and multiantigen models on both platforms against each other, against the serological tests evaluated by the WHO in 2008, and against the study and TPP targets (Figure 4). The 3-antigen models performed significantly better than single antigens and previously evaluated tests by the WHO, but multiantigen combinations did not further improve performance. No model on either platform approached the study or TPP targets.

## DISCUSSION

We carried out a prospective diagnostic study with more than 750 HIV-uninfected adult patients with presumed tuberculosis in high-volume, urban settings in 2 high-burden countries and with 2 multiplexed antibody detection platforms: a field-based platform (MBio) and a reference laboratory platform (Luminex). Given the heterogeneous nature of antibody responses in tuberculosis, we used a large pool of antigens selected from the immunoproteome screen [6] and evaluated their individual performance, as well as the predictive abilities of combinations. Although 3-antigen and multiantigen models performed better than single antigens, our robust and systematic approach showed that no combination met performance targets for tuberculosis detection tests or tuberculosis triage tests on either platform.

The antigens that performed best in our study have also performed well in other studies reported in the literature. In keeping with the fact that antigens were selected from the whole proteome screen [6], 20 of the 22 top antigens from this study were highly ranked in the whole proteome screen, in Khan et al [9], or in both (Supplementary Table 8). The secreted glycoprotein Rv1860 (MPT32, Apa) is present in immunodominant fractions of *M. tuberculosis* H37Rv culture filtrate [15] and reached the highest odds ratio in the *M. tuberculosis* proteome screen [6], as well as in other studies [7, 9]. The 16-kDa cell-wall protein Rv2031c (HspX) was also identified in the proteome screen [6, 16]. Rv1886c, a protein of the antigen 85 (Ag85) complex, is involved in cell wall mycolylation and is immunodominant [9, 16]. A native preparation of the Ag85 complex and an additional production batch of Rv1886c were used in the Luminex assay and also performed well. Only 2 antigens from the Top 22 were not previously identified: a succinate dehydrogenase (Rv3319) and fructose 1,6-bisphosphatase (Rv1099), which is involved in gluconeogenesis [17] and was elevated in infected Rhesus macaques in a tuberculosis outbreak [18]. Superior performance of serological tests has been reported in other studies, although the comparison groups were often asymptomatic patients rather than patients with presumptive tuberculosis subsequently classified as non-tuberculosis disease [19–22]. Differences between our results and those of previous studies could also be attributable to patient population, design, and sample size. While previous studies have often used single-center recruitment, retrospective designs, and smaller sample sizes, our study included prospective consecutive recruitment of patients with presumptive tuberculosis in two high tuberculosis prevalence countries. In the assessment of study quality using the QUADAS-2 framework, our study has a relatively low risk of bias (Supplementary Table 12). In contrast, the metaanalysis of Steingart et al [23] identified “very serious limitations” and “significant risk of bias” for most of the studies, which might explain overestimation of test performance. Using the same search terms as Steingart et al, restricted to adults and pulmonary tuberculosis, we identified 2 publications through PubMed since their metaanalysis that evaluated patients with presumptive tuberculosis [7, 24]. One was a diagnostic study with reported sensitivity of 35.6% and specificity of 93.7% [24], which is similar to our results.

Our study has several limitations. Performance differences between the 2 platforms (Figure 3A) may be due to a degree of variability introduced by day-to-day field testing vs frozen sample batch testing in a research laboratory. However, platform AUC differences were only significant for 5 antigens (Supplementary Table 5), and MBio showed reasonable AUC agreement across sample types (Figure 3A). We recognize that expression systems and antigen purity matter and, therefore, included native antigen preparations and, where available, several versions of the same antigen from multiple suppliers on the Luminex platform. Notably, five of the highest-performing antigens on Luminex were native protein preparations that were added after the MBio platform assay was finalized. However, these showed only marginal improved performance over other antigens (eg, best performing native protein preparation on Luminex, WCL H37Rv_6, AUC = 0.72 vs best performing single antigen on Luminex, Rv2031c_1a, AUC = 0.69). The high performance of native proteins from *M. tuberculosis* strains compared to proteins expressed in *Escherichia coli* may be explained by different post-translational modifications. Expression of selected antigens in *Mycobacterium smegmatis* might therefore be advantageous [25, 26]. The mode of antigen display may also influence antibody binding. However, we could only see marginal differences in diagnostic performance when we used two independent immobilization technologies (antigen printing on plastic substrates and covalent antigen coupling to beads).

As noted in previous IgG biomarker studies, there is remarkable patient-to-patient variability in antibody responses [5–7, 9]. We found the same heterogeneity in individual responses and also that performance of 7 of the 22 high ranked antigens were significantly associated with study location (Figure 3B). Differences in the referral pathways, genetic diversity among tuberculosis strains, host genetic differences [27], differing vaccination status, or exposure to environmental mycobacteria might explain some of this variability [7]. Patients being at different stages of the spectrum of latent to active tuberculosis disease could also contribute [28–30]. Latent tuberculosis infection was not determined by tuberculin skin test or interferon gamma release assay in our study, which is a limitation. Categorized radiological interpretations of the chest radiographs were not available; therefore, we could not analyze data based on cavitary status. Subanalyses excluding patients with a prior history with tuberculosis showed comparable results to the whole dataset. Our findings cannot be applied to children, people living with HIV with presumptive tuberculosis, or to systematic screening or active case-finding.

Our results confirm that a defined pool of antigens are recognized by the IgG antibody response during active tuberculosis and that no single antigen or combination is sufficient to clearly identify patients with active tuberculosis disease [6, 7, 9, 16, 31, 32]. The results of our study of HIV-uninfected patients with presumptive tuberculosis from high-volume settings in tuberculosis endemic countries, which evaluated multiple antigens, sample types, and 2 test platforms, suggests that a conventional antigen-based IgG detection test is unlikely to meet requirements for novel tuberculosis diagnostics in symptomatic patients. This is in concordance with systematic reviews and metaanalyses of serologic testing in tuberculosis [3, 4], although combination with other biomarkers and IgG properties such as FcR-binding and glycosylation characteristics may have potential [33, 34].

## Supplementary Data

Supplementary materials are available at *Clinical Infectious Diseases* online. Consisting of data provided by the author to benefit the reader, the posted materials are not copyedited and are the sole responsibility of the author, so questions or comments should be addressed to the author.

## Supplementary Material

Supplementary_Material

## Notes

***Acknowledgments*.** We thank the study teams in Peru (Universidad Peruana Cayetano Heredia, Lima) and Vietnam (Pham Ngoc Thach Hospital, Ho Chi Minh City) for their efforts to complete data collection in time; Antigen Discovery Inc (ADI), Statens Serum Institut (SSI), Infectious Disease Research Institute (IDRI), Lionex, and Cornell University for providing the antigens; and Nora Champouillon (FIND) for her logistic support.

***Author contributions*.** M. D. P., T. B., and C. M. G. designed the study. M. L. G. and M. D. P. contributed knowledge on the seroreactive immunoproteome of *M. tuberculosis*. I. R., P. A., and M. S. provided recombinant antigens. M. J. L., C. H. G., and G. M. H. led the assay and cartridge development. A. F., S. R., N. S., and T. J. did the Luminex-related work. J. H., D. D., A. F., C. M. D., and T. B. analyzed the data. R. B., T.B., C. M. D., M. L. G., and M. D. P. wrote the manuscript and all authors contributed to and approved the final version.

***Financial support*.** This work was supported by FIND, the Bill & Melinda Gates Foundation (grant OPP1018924), and the Ministry of Foreign Affairs, Government of the Netherlands (grant 22211).

***Potential conflicts of interest*** T. B., C. M. G., J. H., C. M. D., and M. D. P. are employed by FIND (Geneva, Switzerland), a nonprofit organization that collaborates with industry partners. R. B., D. D., and M. L. G. were consultants for FIND. A. F., S. R., N. S., and T. J. are employed by the Natural and Medical Sciences Institute at the University of Tübingen (Reutlingen, Germany), and work was funded by FIND. M. J. L. is chief technology officer (CTO) and C. H. G. and G. M. H. are employees of the commercial partner MBio Diagnostics and received funding from FIND. I. R. and P. A. (employees of the SSI) and M. S. (CEO from Lionex) provided recombinant antigens and were not involved in study design and data analysis. All other authors: No potential conflicts of interest. The authors have submitted the ICMJE Form for Disclosure of Potential Conflicts of Interest. Conflicts that the editors consider relevant to the content of the manuscript have been disclosed.
