# Supplementary material for: Diagnostic Performance of Tuberculosis-Specific IgG Antibody Profiles in Patients with Presumptive Tuberculosis from Two Continents
Source: Clin Infect Dis. 2017 Jan 25;64(7):947–55. doi: 10.1093/cid/cix023 (PMC5848306; doi:10.1093/cid/cix023)
Supplement: Supplementary_Material [file cix023_supp1_supplementary_material.docx]

**Supplementary materials for:**

# Diagnostic performance of tuberculosis-specific IgG antibody profiles in patients with presumptive TB from two continents

Tobias Broger, M.Sc., Robindra Basu Roy BMBCh, Angela Filomena M.Sc., Charles H. Greef PhD, Stefanie Rimmele PhD, Joshua Havumaki M.Ph, David Danks PhD, Nicole Schneiderhan-Marra PhD, Christen M. Gray M.Ph, Mahavir Singh PhD, Ida Rosenkrands PhD, Peter Andersen DMSc, Gregory M. Husar, Thomas O. Joos PhD, Maria L Gennaro M.D., Michael J. Lochhead PhD, Claudia M. Denkinger M.D., and Mark D. Perkins M.D.

Supplementary text, Appendix 1-10: page 3

Table S1: page 16

Table S2: page 17

Table S3: page 18

Table S4: page 23

Table S5: page 24

Table S6: page 25

Table S7: page 26

Table S8: page 27

Table S9: page 28

Table S10: page 29

Table S11: page 30

Table S12: page 31

Figure S1: page 32

Figure S2: page 33

**Supplementary Text:**

**Appendix 1. Subjects excluded from analysis**

Subjects with two contaminated or missing cultures; speciation missing or mixed nontuberculous mycobacteria/*M. tuberculosis*; a single scanty culture-positive; smear-positive, culture-negative patients; Xpert-positive, culture-negative patients; positive at follow-up only, MBio or smear not done; or at least one SE/WB/FZ sample or clinical data missing, were excluded from analysis.

**Appendix 2. Handling of sputum and blood samples**

Smear microscopy was carried out with the Ziehl-Neelsen method, and the remainder processed with N-acetyl-L-cysteine-sodium hydroxide for testing with Xpert MTB/Rif (Cepheid, US), solid culture on Löwenstein-Jensen medium, and liquid culture (BACTEC MGIT 960 culture, BD, US).

Tests on diluted whole blood and serum were carried out the same day on the MBio platform. If a repeat test was needed, serum was stored at 4°C overnight and tested the following day. At least two serum aliquots were frozen at -70°C; one for frozen serum (FZ) testing on MBio and one for shipment to Natural and Medical Sciences Institute (NMI), Germany for Luminex testing.

**Appendix 3. Antigen selection**

Figure S1 summarizes the antigen selection process that led to the selection of the 132 Luminex reference platform antigens and the 57 MBio field platform antigens used in this study. Based on several secondary analyses after publication of the immunoproteome study by Kunnath-Velayudhan et al. [1] the project consortium selected 62 antigens for expression and purification. Included antigens had either a high odds ratio (all proteins from Table 1 in Kunnath-Velayudhan et al.), AUC’s >0.60 or a high random forest model importance. Overall, the consortium applied a low threshold for inclusion to minimize the risk of omitting potentially relevant antigens. 55/62 antigens were successfully expressed in *E.coli* and purified, however 7 of the targets could initially not be purified (4 of them could be recovered later and were included in the Luminex assay of this study, see below and Figure S1). 16 of the antigens were expressed as a second version (same Rv number but in a different production batch) leading to a total of 71 purified antigens originating from the whole proteome screen purified by Antigen Discovery Inc., USA (ADI, indicated with index=1 after the Rv number). To further minimize the risk of missing relevant antigens due to the limitations of the immunoproteome approach, FIND reached-out to institutes (identified via publications, patents or FIND network) with relevant antibody detection expertise to source promising antigens. Four institutes made their materials available under material transfer agreements and the following antigens were included: 21 antigens from Infectious Disease Institute (IDRI, index=2), USA; 13 antigens from Statens Serum Institut (SSI, index=3), Denmark; 4 antigens from Lionex GmbH (index=4), Germany; 3 antigens from Cornell University (index=7), USA. Using the resulting 112 antigens, the NMI Natural and Medical Sciences Institute at the University of Tübingen (Reutlingen, Germany) established a Luminex assay for the purpose of down-selecting antigens for the MBio field platform as described in Rimmele 2012 [2]. The 57 antigens with the highest AUC’s were eventually selected for the MBio field platform and used in this study. For the Luminex reference assay used in this study, we retained all except ten antigens of the original Luminex assay from Rimmele 2012 [2] and added another 30 antigens leading to the 132 antigens for the Luminex assay in this study. The ten exclusions were based on low performance of antigens present in multiple versions. The 30 additions include: 7 native protein preparations from BEI Resources (index=6, for the purpose of comparing the performance of native proteins to recombinant proteins), 19 antigens from new production batches of already included antigens (18 from FIND (index=5) and 1 from ADI (index=1) for the purpose of comparing different production batches) and 4 antigens from ADI (index=4) which could initially not be purified but finally recovered for this study. We identify single antigens by standard Rv nomenclature (<http://tuberculist.epfl.ch/>). The relatively low thresholds for inclusion in combination with antigens provided by collaborating institutes resulted in different versions for some antigens having the same Rv number (see Table S3). Furthermore some of the antigens were produced using different expression systems (other than *E. coli,* see Table S3). Overall, we adopted a comprehensive and inclusive approach to also include antigens based on findings of collaborating institutes and minimized the chance of omitting antigens by applying very low thresholds for inclusion.

**Appendix 4. Antigen expression, purification and quality**

ADI (index=1) and FIND (index=5) antigens were produced using standard *E. coli* expression systems using pXT7 plasmid vectors and purification by affinity chromatography (using the co-expressed GST or His-tag of the protein). A number of targets could not be expressed initially and alternative plasmid vectors (pET24d and pGEX2T-N-His) were used. All recombinant antigens (including antigens from collaborating institutes IDRI, SSI, Lionex, Cornell, ADI, FIND) were shipped to NMI (Reutlingen, Germany) and the expression yield and the level of purity was monitored by SDS-PAGE and subsequent Coomassie staining as a quality control prior to bead coupling. >95% purity according to visualization of SDS-PAGE gels was defined as acceptable. ≥0.1 mg was the minimum required amount for this study. In total about 70% of all recombinant antigens showed good (only minor additional protein bands visible on the SDS gel) to excellent (no additional protein bands visible on the SDS gel) purity. Native protein preparations (from BeiResources) were not run on SDS gels. Further details on the cloning and expression of antigens as well as images of gels, including for antigens from collaborating institutes, are available upon request.

**Appendix 5. MBio multiplexed serological platform:**

The MBio system uses fluorescence illumination in which a laser is directed into a planar waveguide by an integrated lens, causing excitation and emission of fluorescently labelled probes on the waveguide’s surface [3]. Each cartridge incorporates a two-dimensional array of antigens and controls spots into a simple fluidic channel, the base of which is the optical waveguide.

The assay was designed to include procedural and data normalization controls, including fiducial marking spots (“Fiducial Spots”) at the outside position of each row, buffer-only spots in each row to allow background subtraction (called “Negative Control Spots” below), and a general anti-human IgG capture antibody for procedural control verification. The fiducial markers serve a dual purpose of providing spot finding anchors for the image analysis software, as well as providing reference signal for row-to-row signal intensity normalization.

The analysis software delivers a normalized value scaled to an equalized row-wise signal output. Details of the algorithm are proprietary and are not disclosed here, but the general concept is described by the following equation:

*(Raw Spot Intensity – Negative Control Spot Intensity)*

*(Fiducial Spot Intensity – Negative Control Spot Intensity)*

*Normalized Spot Intensity =*

The “Normalized Spot Intensity” is the value reported from the SnapEsi reader. Tests confirmed that the method equalizes row-wise signal intensities within 5% variation (data not shown).

Array data analysis was performed after raw data was uploaded from the study sites to the FIND FTP server in Geneva. MBio performed image analysis as FIND released blinded aggregated raw data.

MBio data analysis was done using raw signals, although for some visualizations (such as for boxplots), MBio raw data was increased by 1 and then log transformed (log(raw intensity +1)). This transformation was only used for visualization purposes due to the fact that the MBio raw dataset contained values below 0 and is mentioned in the respective figures. For visualization of antigen reactivity in heatmaps standard z-scores were calculated based on log transformed values. Raw data are available upon request.

**Appendix 6. Luminex bead-based serological platform**

The Luminex xMAP technology is a multiplexed technology using colour-coded polystyrene microspheres (6.5 μm in diameter) and the principles of flow cytometry as the readout technology. Each microsphere is impregnated with up to ten different concentrations of three internal dyes (red, infrared and orange), which can be accurately discriminated by the Luminex instrument. A red laser and three different photomultipliers classify the internal dyed microspheres and a green laser excites the fluorescence-labelled reporter molecules that detect antigen bound IgG. Using the colour-coding, up to 500 microspheres can be individually distinguished. Antigens were covalently coupled to colour-coded beads (MagPlex Microspheres, Luminex Corp, Austin, TX) using ECD/Sulfo-NHS chemistry and an automated bead handler (KingFisher96, Thermo Scientific, Schwerte, Germany). All recombinant antigens (n=125) were expressed with a His-tag and coupling efficiency of the antigens to the Luminex beads was confirmed using an anti-His antibody and secondary R-PE conjugated anti-species antibody for detection of bead-bound antigen.

In addition to the patient samples, each 96 well plate includes one (non-endemic) negative control from healthy donors and a positive control sample. The positive control sample is systematically generated thorough the combination of 3 positive sera and shows reactivity to most antigens.[4] Assay validation was performed according to assay validation guidelines.[5–7]

For the bead-based serological assay to detect IgG antibodies, frozen sera were thawed and diluted in assay buffer (1:200, PBS + Low Cross Buffer (Candor, Wangen, Germany) + 5 g/L BSA) supplemented with 10 % *E. coli* lysate. After incubation (20 min on a shaker) 50 µL of diluted serum sample was mixed with master bead mix (antigen coupled microspheres, for each antigen at least 100 beads per well), and incubated for 2 h at 20°C using the automated bead handler. Unbound antibodies were removed by washing the beads twice with 100 µL washing buffer (PBS + 0.05 % Tween20). To visualize antigen-bound human IgG the beads were incubated (1 h, 20°C) with 50 µL of an R-PE labeled goat anti-human IgG antibody (5 µg/mL, Jackson Dianova, Hamburg, Germany) diluted in assay buffer. After washing twice with 100 µL washing buffer the beads were resuspended in 100 µL washing buffer. Readout was performed using a Luminex FlexMAP3D instrument (Luminex Corp, Austin, TX) and stopped after 80 µL were measured. Binding events were displayed as median fluorescence intensity (MFI) based on >60 measured beads per bead sort and used for final data analysis.

*Assay validation*

Assay validation was performed according to assay validation guidelines from the FDA (Food and Drug Association) and EMA (European Medicines Agency). Intra-assay coefficients of variation (CVs) were calculated per antigen based on the measurement of eight replicates of the same sample (in total 6 selected serum samples from active TB patients were required to ensure sufficient signal above the background for every antigen). Intra-assay CVs were <20% for all except one antigen (131/132). For inter-assay variation the experiment was repeated three times within one week by running triplicates of each sample resulting in inter-assay CVs <20% for all except three antigens (129/132).

*Signal normalization*

Luminex raw MFI data were log transformed prior to all analysis and visualization. Raw data are available upon request.

**Appendix 7. MBio – Luminex Concordance: Phase I Analysis**

The MBio cartridge used in this study was designed to simultaneously measure 57 antigen-antibody interactions in a clinical sample. Because this was an unprecedented degree of multiplexing in a field-portable platform, the MBio assay was first validated against Luminex in Study Phase I with 218 patients (184 valid results) in Peru and Vietnam [8]. The two platforms (MBio and Luminex) yield outputs on different scales, and so concordance was based on the z-scores of IgG reactivity (fluorescence signal intensity). The z-score of a particular signal intensity is the number of standard deviations (SD) it is above or below the average signal of all samples for a specific antigen. Z-scores were used to categorize the data as: negative (significantly below the average), neutral (not significantly different from the average), and positive (significantly above the average). A threshold of 1 SD was used to divide the data into positive, neutral, and negative. We then computed the percent agreement (concordance) for every antigen. Concordance between the platforms on this basis was good, ranging from 76.1% to 99.5% for the entire antigen set. 11 (Rv1860_1a, Rv2031c_1a, Rv3875_4, Rv3881c_1a, Rv0934_1a, Rv1196_1c, Rv3616_1e, Rv0798c_1b ls, Rv1099_2, Rv2873_1f, Rv2875_2) of the 22 best performing antigens showed very high concordance (>95%) and only one of the 22 best performing antigens showed a concordance <80% (Rv1886_2, 77.2%).

**Appendix 8. Diagnostic performance of single antigens and models**

The study targets were defined slightly below those of the respective TPP as during the final stages of product development, performance can often be further improved. Two signal intensity cut-offs were defined based on study targets:

(i) for TB detection test the signal intensity cut-offs leading to a specificity of 90% for MBio and 95% for Luminex respectively were used and the corresponding sensitivity at pre-set specificity was calculated and reported as “TB detection test sensitivity”.

(ii) for TB triage test the signal intensity cut-offs leading to a sensitivity of 85% for MBio and 90% for Luminex respectively were used and the corresponding specificity at pre-set sensitivity was calculated and reported as “TB triage test specificity”.

In addition to TB detection test sensitivity at pre-set specificity and TB triage test specificity at pre-set sensitivity Area Under the Curve (AUC) were calculated and used for a direct comparison of antigen performance between platforms (MBio field platform vs. Luminex reference platform) and study groups (per geography, TB history). For all performance, measures 95% confidence intervals (CIs) were calculated. 95% CIs for AUCs were calculated using a logit, non-parametric confidence intervals estimator.[9]

**Appendix 9. Statistical software**

SAS 9.3 was used for univariate analysis. Matlab2014a was used to create boxplots, ROC curves and heatmaps. The classification models were implemented in the statistical program R (version 3.1, see www.r-project.org), using functionality included in the packages nnet, e1071, gam, and ROCR available on CRAN (www.cran.r-project.org).

**Appendix 10. Statistical analysis for multi-antigen combinations**

Generalized linear models (GLMs) and generalized additive models (GAMs) predict TB status using the weighted sum of antibody levels to different antigens, where the latter first transform the antibody levels by a smooth function to emphasize critical (according to the data) values. In addition, several classification approaches to classify TB status based on antibody levels were tested. Naïve Bayes (NB) models performed best. Three different performance measures were used for model tuning: sensitivity at pre-set specificity (TB detection test), specificity at pre-set sensitivity (TB triage test) and optimization of AUC. I.e. sensitivity was maximized subject to a specificity fixed at 0.9 for the TB detection test (0.95 for the Luminex data) and specificity subject to a sensitivity fixed at 0.85 for the TB triage test (0.9 for Luminex data).

*Three-antigen models*

For all sample types (WB, SE and FZ for MBio and FZ for Luminex) models were trained separately on all possible three-antigen combinations of the 57 antigens (29,260 input sets in total) using 8-fold cross-validation, and then found the three-antigen input sets that achieved the best TB detection test sensitivity, TB triage test specificity and AUC. We further examined whether certain antigens were frequently part of good-performing subsets by counting the members of the ten best-performing triples for each performance measure. Finally, we compared the frequency of an antigen in the top ten models to its performance as a single antigen (Table S7).

*Multi-antigen models stepwise regression*

The stepwise regression was started with the set of all antigens and antigens were repeatedly removed based on which removal provided the greatest decrease in Akaike Information Criterion (AIC), a measure of the model’s fit to the data. For each reduced antigen set, 8-fold cross-validation with the six different types of models was used, including two different Neural Networks (NNs, with 3 vs. 5 hidden units) and Support Vector Machines (SVMs, whereas a Gaussian radial basis function kernel and a constant cost parameter was used as a different measure of variable importance).

*N-fold cross validation*

In this technique, data are divided into N subsets of roughly equal size and properties (e.g., similar numbers of TB+ individuals) called folds. For a given model-type and dataset, N different models were trained, each on all-but-one of the folds. Subsequently each model was tested on the fold that was not used in its training to estimate the performance.

**References**

1. Kunnath-Velayudhan S, Salamon H, Wang H-Y *et al.* Dynamic antibody responses to the Mycobacterium tuberculosis proteome. *Proceedings of the National Academy of Sciences* 2010;**107**:14703–8.

2. Rimmele S. Dissertation: Mycobacterium tuberculosis Antigen Arrays for the in-depth Characterization of the Serological Response of TB Infected Patient Cohorts. 2012.

3. Lochhead MJ, Todorof K, Delaney M *et al.* Rapid multiplexed immunoassay for simultaneous serodiagnosis of HIV-1 and coinfections. *Journal of Clinical Microbiology* 2011;**49**:3584–90.

4. Planatscher H, Rimmele S, Michel G *et al.* Systematic reference sample generation for multiplexed serological assays. *Scientific Reports* 2013;**3**:3259.

5. Jacobson RH. Validation of serological assays for diagnosis of infectious diseases. *Revue scientifique et technique (International Office of Epizootics)* 1998;**17**:469–526.

6. Food and Drug Administration. *Guidance for Industry Bioanalytical Method Validation*., 2001.

7. European Medicines Agency. *Guideline on Validation of Bioanalytical Methods. (EMEA/CHMP/EWP/192217/2009)*., 2009.

8. Greef C, Husar G, Gray C *et al.* Highly Multiplexed Detection of Antibodies in Whole Blood during Tuberculosis Infection. *21st Conference on Retroviruses and Opportunistic Infections*. Boston, MA, USA, 2014.

9. Gengsheng Qin, Hotilovac L. Comparison of non-parametric confidence intervals for the area under the ROC curve of a continuous-scale diagnostic test. *Statistical Methods in Medical Research* 2008;**17**:207–21.

10. Khan IH, Ravindran R, Krishnan V V *et al.* Plasma antibody profiles as diagnostic biomarkers for tuberculosis. *Clinical and Vaccine Immunology* 2011;**18**:2148–53.

**Supplementary Table S1: Inclusion and exclusion criteria for entry into the trial.**

| **Inclusion criteria** | **Exclusion criteria** |
| --- | --- |
| Symptoms suggesting pulmonary TB, i.e. persistent cough (generally > 3 weeks or as per local definition of TB suspect) and at least one other finding listed below:   - Fever - Malaise - Recent weight loss - Night sweats - Contact w/ active case - Haemoptysis - Chest pain - Loss of appetite | Patients receiving any anti-tuberculosis medication, including fluoroquinolone and aminoglycosides in the 60 days prior to enrolment. |
| Provision of informed consent to sample collection, banking and HIV testing. | Patients with only extra-pulmonary disease. |
| Production of two sputum samples of at least 1·5 mL and consent to blood samples for serum and whole blood tests | Patients for whom good follow-up and a clear final diagnosis are judged to be difficult |
| Adult age (>18 years old) | HIV positive |

Supplementary Table S2: Tuberculosis case definitions. (LJ= Löwenstein–Jensen solid medium, MGIT= BACTEC Mycobacteria Growth Indicator Tube 960 culture, NTM=Non-Tuberculous Mycobacteria, MTB=*M. tuberculosis*)

| Definite TB | Culture-positive (TB+) | ≥ 1 LJ and/or MGIT culture growth confirmed MTB complex.  Cross-Contamination: A single LJ culture with ≤ 20 colonies or a single MGIT culture with MTB growth ≥28 days per patient (excluded from analysis).  NTM: Specimens with growth of mycobacteria other than MTB complex only (excluded from analysis). |
| --- | --- | --- |
|  | Smear-positive,  Culture-positive (S+C+) | Culture positive and ≥ 1+ smear (≥10/100 fields) or ≥ 2 scanty-positive smears. |
|  | Smear-negative, Culture-positive (S-C+) | Culture positive and all smears negative or only a single scanty-positive smear. |
| Clinical TB | Smear-negative, Culture-negative (S-C-), started on empiric TB treatment and responded | Neither LJ nor MGIT has culture growth after >56 days and >42 days, respectively and at least 2 cultures have no signs of contamination. All smears negative. |
| NTBD | Not-TB disease (TB-) | A subset of S-C-. Smear negative, culture-negative patient, chest x-ray not indicative of TB, symptoms resolving at 2-month follow-up without TB treatment based on clinical criteria. |

**Supplementary Table S3: Antigen list**

| **Rv number** | **Supplier** | **Index** | **Name** | **Luminex** | **MBio** | **Remark** |
| --- | --- | --- | --- | --- | --- | --- |
|  |  |  |  |  |  |  |
| NA | beiResources | 6 | Ag85 complex_6 | **Y** |  | Purified Native Protein from *Mycobacterium tuberculosis*, Strain 37Rv |
| NA | beiResources | 6 | CFP H37Rv_6 | **Y** |  | Culture Filtrate Proteins *Mycobacterium tuberculosis* |
| NA | beiResources | 6 | Cytosol H37Rv_6 | **Y** |  | Cytosol Fraction *Mycobacterium tuberculosis* |
| NA | beiResources | 6 | Cytosol Indo_6 | **Y** |  | Strain Indo-Oceanic T17X, Cytosol Fraction, *Mycobacterium tuberculosis* |
| NA | beiResources | 6 | Tx-114 H37Rv_6 | **Y** |  | TX-114 Soluble Proteins *Mycobacterium tuberculosis* |
| NA | beiResources | 6 | WCL H37Rv_6 | **Y** |  | Whole Cell Lysate *Mycobacterium tuberculosis* |
| NA | beiResources | 6 | WCL Indo_6 | **Y** |  | Strain Indo-Oceanic Whole Cell Lysate *Mycobacterium tuberculosis* |
| NA | Lionex | 4 | Antigen Cocktail V_4 | **Y** |  | Recombinant antigen cocktail, 4 antigens |
| Rv2031c-Rv2873 | SSI | 3 | Rv2031c-Rv2873_3 | **Y** |  | Fusion protein Rv2031c-Rv2873 |
| Rv3874-Rv3875 | IDRI | 2 | C10-E6_2 | **Y** |  | C10-E6, complex of Rv3874-Rv3875 (cfp10-esat-6, esxB-esxA) |
|  | SSI | 3 | Rv3874-Rv3875_3 | **Y** |  | C10-E6, complex of Rv3874-Rv3875 (cfp10-esat-6, esxB-esxA) |
| Rv0379-Rv0934-Rv3874 | IDRI | 2 | TBF10_2 | **Y** |  | Fusion protein Rv0379-Rv0934-Rv3874, TBF10 |
| Rv2031-Rv0934-Rv3874 | IDRI | 2 | DID64_2 | **Y** |  | 64 kDa fusion protein Rv2031-Rv0934-Rv3874 |
| Rv2031-Rv0934-Rv2032 | IDRI | 2 | DID90_2 | **Y** |  | 90 kDa fusion protein Rv2031-Rv0934-Rv2032 |
| Rv0212 | ADI | 1 | Rv0212_1b ss | **Y** |  |  |
| Rv0222 | SSI | 3 | Rv0222_3 | **Y** | **Y** |  |
| Rv0272 | ADI | 1 | Rv0272_1b ss | **Y** | **Y** |  |
| Rv0302 | ADI | 1 | Rv0302_1c | **Y** | **Y** |  |
| Rv0379 | ADI | 1 | Rv0379_1a | **Y** | **Y** |  |
| Rv0394 | ADI | 1 | Rv0394_1c | **Y** |  |  |
| Rv0440 | ADI | 1 | Rv0440_1d | **Y** | **Y** |  |
| Rv0456 | ADI | 1 | Rv0456c_1a | **Y** | **Y** |  |
| Rv0577 | SSI | 3 | Rv0577_3 | **Y** | **Y** |  |
|  | Cornell | 7 | Rv0577 *E. coli*_7 | **Y** |  |  |
|  | Cornell | 7 | Rv0577 *Pichia*_7 | **Y** |  | Expressed in *Pichia pastoris* |
|  | Cornell | 7 | Rv0577 BCG_7 | **Y** |  | Expressed in BCG |
|  | FIND | 5 | Rv0577_5 | **Y** |  |  |
| Rv0583 | ADI | 1 | Rv0583c_1c | **Y** | **Y** |  |
| Rv0632 | ADI | 1 | Rv0632c_1c | **Y** | **Y** |  |
| Rv0798 | ADI | 1 | Rv0798c_1b ls | **Y** | **Y** |  |
| Rv0801 | ADI | 1 | Rv0801_1b ls | **Y** | **Y** |  |
| Rv0831 | IDRI | 2 | Rv0831_2 | **Y** |  |  |
| Rv0934 | ADI | 1 | Rv0934_1a | **Y** | **Y** |  |
|  | IDRI | 2 | Rv0934_2 | **Y** |  |  |
| Rv0944 | ADI | 1 | Rv0944_1d | **Y** | **Y** |  |
| Rv0984 | ADI | 1 | Rv0984_1c | **Y** | **Y** |  |
| Rv1009 | IDRI | 2 | Rv1009_2 | **Y** | **Y** |  |
| Rv1030 | ADI | 1 | Rv1030_1f | **Y** |  |  |
| Rv1030extern | FIND | 5 | Rv1030extern_5 | **Y** |  | Extracellular region |
| Rv1030intra | FIND | 5 | Rv1030intra_5 | **Y** |  | Intracellular region |
| Rv1099 | IDRI | 2 | Rv1099_2 | **Y** | **Y** |  |
| Rv1175c | ADI | 1 | Rv1175c_1f | **Y** |  |  |
|  | FIND | 5 | Rv1175_5 | **Y** |  |  |
| Rv1196 | ADI | 1 | Rv1196_1c | **Y** | **Y** |  |
| Rv1242 | ADI | 1 | Rv1242_1b ls | **Y** | **Y** |  |
| Rv1284 | ADI | 1 | Rv1284_1a | **Y** | **Y** |  |
| Rv1387 | ADI | 1 | Rv1387_1g | **Y** |  |  |
| Rv1411c | ADI | 1 | Rv1411c_1a | **Y** | **Y** |  |
| Rv1566c | ADI | 1 | Rv1566c_1a | **Y** | **Y** |  |
| Rv1586c | SSI | 3 | Rv1586c_3 | **Y** |  |  |
| Rv1629 | ADI | 1 | Rv1629_1f | **Y** |  |  |
|  | FIND | 5 | Rv1629_5 | **Y** |  |  |
| Rv1636 | SSI | 3 | Rv1636_3 | **Y** | **Y** |  |
| Rv1837c | ADI | 1 | Rv1837c_1c | **Y** | **Y** |  |
| Rv1860 | ADI | 1 | Rv1860_1a | **Y** | **Y** |  |
|  | IDRI | 2 | Rv1860_2 | **Y** |  |  |
|  | Lionex | 4 | Rv1860_4 | **Y** |  |  |
| Rv1886c | ADI | 1 | Rv1886c_1f | **Y** |  |  |
|  | IDRI | 2 | Rv1886_2 | **Y** | **Y** |  |
|  | FIND | 5 | Rv1886_5 | **Y** |  |  |
| Rv1926c | ADI | 1 | Rv1926c_1c | **Y** | **Y** |  |
| Rv1980c | ADI | 1 | Rv1980c_1c | **Y** |  |  |
|  | IDRI | 2 | Rv1980_2 | **Y** | **Y** |  |
|  | SSI | 3 | Rv1980_3 | **Y** |  |  |
| Rv1984c | ADI | 1 | Rv1984_1b ss | **Y** |  |  |
|  | ADI | 1 | Rv1984c_1f | **Y** |  |  |
|  | IDRI | 2 | Rv1984_2 | **Y** | **Y** |  |
|  | SSI | 3 | Rv1984_3 | **Y** |  |  |
| Rv2031c | ADI | 1 | Rv2031c_1a | **Y** | **Y** |  |
|  | IDRI | 2 | Rv2031_2 | **Y** |  |  |
| Rv2032 | IDRI | 2 | Rv2032_2 | **Y** |  |  |
| Rv2094c | ADI | 1 | Rv2094c_1f | **Y** |  |  |
|  | FIND | 5 | Rv2094_5 | **Y** |  |  |
| Rv2151 | ADI | 1 | Rv2151_1d | **Y** | **Y** |  |
| Rv2185c | SSI | 3 | Rv2185c_3 | **Y** |  |  |
| Rv2220 | IDRI | 2 | Rv2220_2 | **Y** |  |  |
| Rv2252 | ADI | 1 | Rv2252 T01_1b ls | **Y** | **Y** |  |
| Rv2282 | ADI | 1 | Rv2282_1b ss | **Y** | **Y** |  |
| Rv2396 | ADI | 1 | Rv2396_1g | **Y** |  |  |
|  | FIND | 5 | Rv2396_5 | **Y** |  |  |
| Rv2462c | SSI | 3 | Rv2462c_3 | **Y** |  |  |
| Rv2544 | ADI | 1 | Rv2544_1d | **Y** | **Y** |  |
| Rv2618 | ADI | 1 | Rv2618_1d | **Y** |  |  |
| Rv2746c | ADI | 1 | Rv2746c_1g | **Y** |  |  |
| Rv2870 | ADI | 1 | Rv2870_1d | **Y** | **Y** |  |
| Rv2873 | ADI | 1 | Rv2873_1f | **Y** | **Y** |  |
|  | IDRI | 2 | Rv2873_2 | **Y** |  |  |
|  | FIND | 5 | Rv2873_5 | **Y** |  |  |
| Rv2875 | ADI | 1 | Rv2875_1f | **Y** |  |  |
|  | IDRI | 2 | Rv2875_2 | **Y** | **Y** |  |
|  | FIND | 5 | Rv2875_5 | **Y** |  |  |
| Rv2927 | ADI | 1 | Rv2927_1a | **Y** | **Y** |  |
| Rv2984 | ADI | 1 | Rv2984_1d | **Y** | **Y** |  |
| Rv3050 | ADI | 1 | Rv3050_1b ss | **Y** | **Y** |  |
| Rv3243 | ADI | 1 | Rv3243_1b ls | **Y** | **Y** |  |
| Rv3248 | ADI | 1 | Rv3248_1a | **Y** | **Y** |  |
| Rv3319 | ADI | 1 | Rv3319_1b ss | **Y** | **Y** |  |
| Rv3326 | ADI | 1 | Rv3326_1b ss | **Y** |  |  |
|  | ADI | 1 | Rv3326_1f | **Y** |  |  |
|  | FIND | 5 | Rv3326_5 | **Y** |  |  |
| Rv3354 | SSI | 3 | Rv3354_3 | **Y** | **Y** |  |
|  | FIND | 5 | Rv3354_5 | **Y** |  |  |
| Rv3362c | ADI | 1 | Rv3362_1b ss | **Y** |  |  |
|  | ADI | 1 | Rv3362c_1f | **Y** |  |  |
|  | FIND | 5 | Rv3362_5 | **Y** |  |  |
| Rv3376 | ADI | 1 | Rv3376_1c | **Y** |  |  |
| Rv3495c | ADI | 1 | Rv3495c_1f | **Y** |  |  |
|  | FIND | 5 | Rv3495_5 | **Y** |  |  |
| Rv3616 | ADI | 1 | Rv3616_1e | **Y** | **Y** |  |
|  | FIND | 5 | Rv3616_5 | **Y** |  |  |
| Rv3628 | ADI | 1 | Rv3628_1d | **Y** | **Y** |  |
| Rv3762c | ADI | 1 | Rv3762c_1c | **Y** | **Y** |  |
| Rv3763 | ADI | 1 | Rv3763_1c | **Y** | **Y** |  |
| Rv3775 | ADI | 1 | Rv3775_1b ss | **Y** | **Y** |  |
| Rv3804c | ADI | 1 | Rv3804c_1a | **Y** |  |  |
|  | IDRI | 2 | Rv3804_2 | **Y** | **Y** |  |
| Rv3810 | ADI | 1 | Rv3810_1g | **Y** | **Y** |  |
|  | FIND | 5 | Rv3810_5 | **Y** |  |  |
| Rv3841 | IDRI | 2 | Rv3841_2 | **Y** | **Y** |  |
| Rv3864 | ADI | 1 | Rv3864_1e | **Y** | **Y** |  |
| Rv3872 | SSI | 3 | Rv3872_3 | **Y** | **Y** |  |
|  | FIND | 5 | Rv3872_5 | **Y** |  |  |
| Rv3874 | ADI | 1 | Rv3874_1a | **Y** | **Y** |  |
|  | IDRI | 2 | Rv3874_2 | **Y** |  |  |
|  | Lionex | 4 | Rv3874_4 | **Y** |  |  |
| Rv3875 | ADI | 1 | Rv3875_1g | **Y** | **Y** |  |
|  | Lionex | 4 | Rv3875_4 | **Y** | **Y** |  |
|  | FIND | 5 | Rv3875_5 | **Y** |  |  |
| Rv3878 | ADI | 1 | Rv3878_1a | **Y** |  |  |
| Rv3879 | SSI | 3 | Rv3879c amino acids 1-181_3 | **Y** |  | Amino Acids 1-181 |
| Rv3881 | ADI | 1 | Rv3881c_1a | **Y** | **Y** |  |
|  | IDRI | 2 | Rv3881_2 | **Y** |  |  |

**Supplementary Table S4: Patient enrolment during the study.**

|  | **Peru** | **Vietnam** | **Total** |
| --- | --- | --- | --- |
| **FIRST PHASE (4 months)** |  |  |  |
| enrolled patients | 67 | 151 | 218 |
| excluded patients | 18 | 16 | 34 |
| valid clinical results | 49 | 135 | 184 |
|  |  |  |  |
| **SECOND PHASE(16 months)** |  |  |  |
| enrolled patients | 374 | 252 | 626 |
| excluded patients | 18 | 37 | 55 |
| valid clinical results | 356 | 215 | 571 |
|  |  |  |  |
| **TOTAL valid results** | **405** | **350** | **755** |

Supplementary Table S5: Overview of top five ranked single antigens (light blue colour) for the measured sample types (serum, whole blood, frozen serum) on the two platforms (MBio and Luminex). The ranking is based on three measures: (a) TB detection test sensitivity and (b) TB triage test specificity and (c) AUC. In total 22 out of the 57 antigens available on both (Luminex reference and MBio field) platforms were ranked as top five in at least one category or sample type. Maximal values and study endpoint targets are included at the bottom of the table (red numbers represent the highest performance in the respective group). AUC’s were not significantly different between sample types. Five (marked §) out of the 22 antigens showed a significantly higher AUC on the reference platform when compared to the performance in frozen sera measured on the field platform, although the absolute differences were small.

**
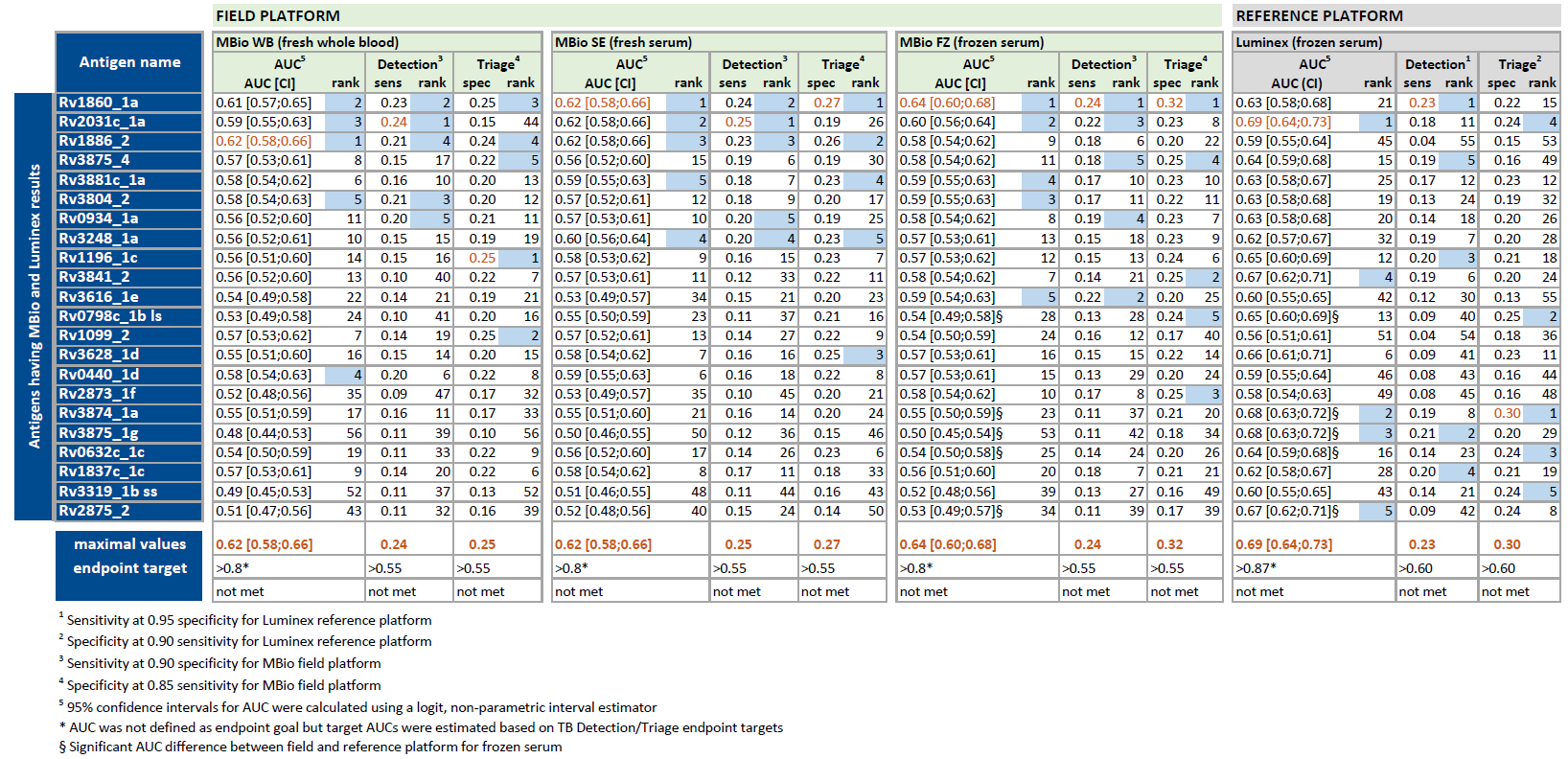
**

Supplementary Table S6: Performance of seven antigens that were only measured on Luminex and performed better than the 57 antigens that were included on both platforms (Table S5). Five of the antigens are native protein preparations and were not available at the time of the antigen down-selection work prior to this study, were therefore not included on MBio but newly added to the Luminex assay for this study as comparators to recombinant antigens. One antigen (Rv1886_5) comes from a new production batch of an already on the MBio platform included antigen (Rv1886_2) that was not available at the time of antigen down-selection. One antigen (Rv3804_1a) was not included on the MBio platform since a similar antigen (Rv3804_2, from a different supplier) was selected for the MBio platform in the down-selection work prior to this study (see Figure S1 and Appendix 3 for further information on antigen selection).


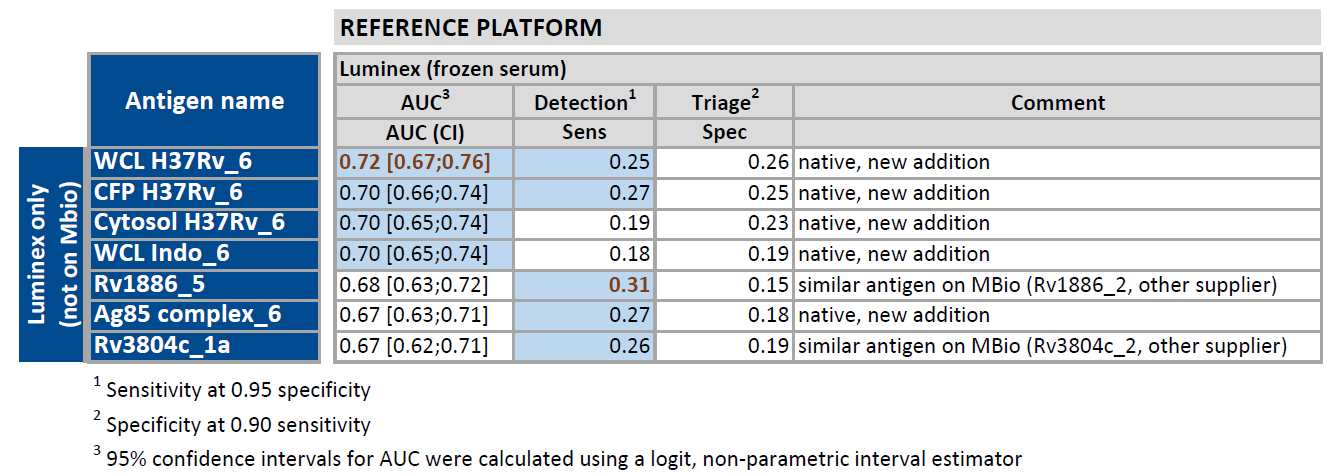


Supplementary Table S7: Frequency of the 22 top ranked single antigens in the (ten) best performing three-antigen models. The ten best three-antigen models were selected on the basis of Naïve Bayes models of all possible three-antigen combinations of the 57 antigens available on both platforms (29260 tested combinations in total). 15 (highlighted in red) out of 22 antigens that showed a high single antigen performance (light blue colour, see also Table S6) also occurred in at least one multivariate model. The table largely overlaps with the best performing single antigens and higher ranked antigens occur more frequently in 3-antigen models. (Det=Detection; Tri=Triage; #=frequency in the ten best three-antigen models; FZ=frozen serum; SE=fresh serum; WB=fresh whole blood)

**
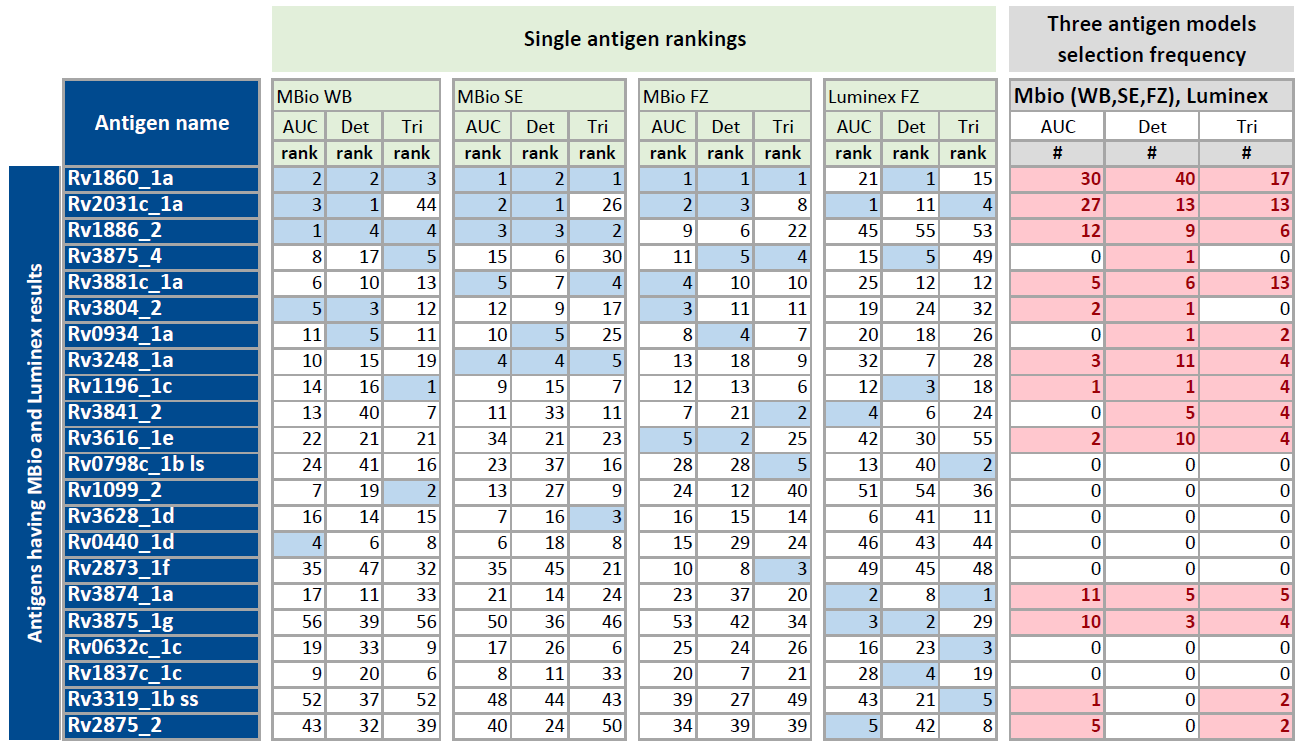
**

Supplementary Table S8: Overlap of the 22 top ranked antigens from this study with highly ranked antigens from Khan et al. 2011 [10] and the whole proteome study from Kunnath-Velayudhan et al. 2010 [1]. In Khan et al. antibodies against tier 1 and tier 2 antigens were at least 3-fold (P value < 0.001) and 1.5-fold (P value > 0.01) higher, respectively, in patients with TB in comparison to the healthy control group. In Kunnath-Velayudhan *et* al, we grouped antigens based on their odds ratio (tier 1: 15-30; tier 2: 5-15; tier 3: 3-5). Kunnath-Velayudhan *et al* reported that Rv3875 (§) was one of the antigens not covered in the overall proteome. This is also the case for Rv1886 and Rv3319 (personal communication with Antigen Discovery Inc.). Rv1099 and Rv3319 are the only antigen that were not identified by these studies.

| **Top 22 antigens this study** | **Khan et al.  2011** | **Kunnath-Velayudhan  et al. 2010** |
| --- | --- | --- |
| Rv1860_1a | Tier 1 | Tier 1 |
| Rv2031c_1a | Tier 1 | Tier 1 |
| Rv1886_2 | Tier 1 | § |
| Rv3875_4 | Tier 2 | § |
| Rv3881c_1a | Tier 1 | Tier 1 |
| Rv3804_2 | Tier 1 | Tier 1 |
| Rv0934_1a | Tier 1 | Tier 1 |
| Rv3248_1a |  | Tier 2 |
| Rv1196_1c |  | Tier 1 |
| Rv3841_2 | Tier 2 |  |
| Rv3616_1e |  | Tier 1 |
| Rv0798c_1b ls |  | Tier 3 |
| Rv1099_2 |  |  |
| Rv3628_1d |  | Tier 3 |
| Rv0440_1d |  | Tier 2 |
| Rv2873_1f |  | Tier 1 |
| Rv3874_1a | Tier 2 | Tier 1 |
| Rv3875_1g | Tier 2 | § |
| Rv0632c_1c |  | Tier 1 |
| Rv1837c_1c |  | Tier 2 |
| Rv3319_1b ss |  | § |
| Rv2875_2 | Tier 2 |  |

Supplementary Table S9: Performance of the top three ranked antigens by study site for MBio frozen serum. Antibody responses were dependent on geographic region (Vietnam, Peru). Antibody response for Rv1860_1a was significantly higher in Vietnam resulting in significantly higher TB detection test sensitivity in Vietnam. Antibody response for Rv2031c_1a and Rv1886_2 was significantly higher in Peru resulting in significant AUC differences and a better TB triage test performance for Rv1886_2.


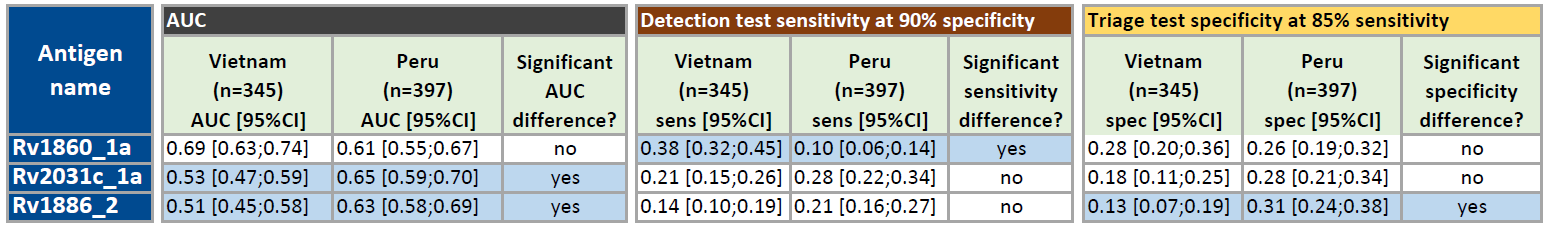


Supplementary Table S10: Impact of TB history on AUC’s for the top ranked 22 antigens. AUC’s were not significantly different compared to all patients when individuals with TB history and TB history unknown were excluded from the analysis (column “no TB History”).


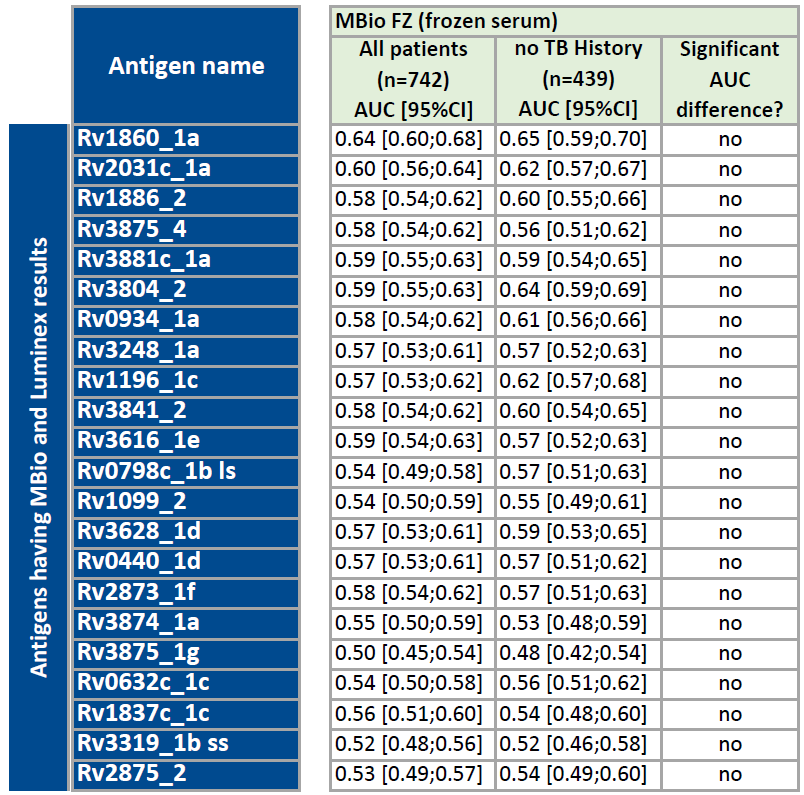


Supplementary Table S11A: Luminex coupling control for the top ranked 22 antigens. All recombinant antigens were expressed with a His-tag and coupling efficiency of the antigens to the Luminex beads was confirmed using an anti-His antibody and secondary R-PE conjugated anti-species antibody for detection of bead-bound antigen. All 22 high performing antigens revealed strong signals close to or above 20000 MFI showing successful coupling of the antigens. Rv3319_1b ss showed relatively high blank signal but still an acceptable S/B ratio allowing for quantitative measurements but within a relatively small dynamic range compared to other antigens. (MFI=Median Fluorescence Intensity)


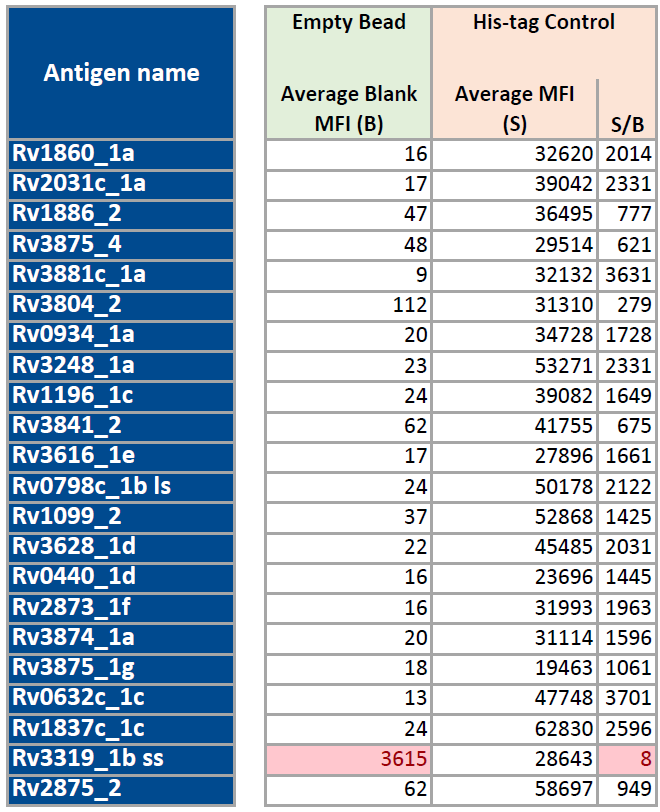


Supplementary Table S11B: Luminex coupling control for all antigens. MFI levels for the 125 his-tagged antigens show successful coupling of all antigens to beads via detection of the His-tag using an anti-His antibody and secondary R-PE conjugated anti-species antibody. (MFI=Median Fluorescence Intensity)

| **His-tag control**  **(MFI levels)** | **# antigens** | **Comment** |
| --- | --- | --- |
| low: <8000 MFI | 0 |  |
| medium: 8000-10000 MFI | 2 | Rv3495_5= 9264 MFI; Rv1629_5= 8670 MFI |
| high: 10000-50000 MFI | 105 |  |
| very high: >50000 MFI | 18 |  |
| Not applicable | 7 | The 7 native antigens have no His-tag |

Supplementary Table S12: QUADAS-2. Self-assessment

| **DOMAIN** | **SIGNALLING QUESTIONS** | **ANSWER** | **DESCRIPTION** |
| --- | --- | --- | --- |
| Patient Selection | Was a consecutive or random sample of patients enrolled? | YES ☺ | Consecutive patient enrollment |
|  | Was a case-control design avoided? | YES ☺ | Cohort study design |
|  | Did the study avoid inappropriate exclusions? | YES ☺ | The study findings are restricted to adults without HIV-infection. HIV-positive adults were excluded |
| Index Test | Were the index test results interpreted without knowledge of the results of the reference standard? | YES ☺ | MBio as well as NMI have sent antibody data to FIND before unblinding. |
|  | If a threshold was used, was it pre-specified? | NO ☹ | Assay and model cut-offs were defined during the data analysis and not pre-specified. |
| Reference Standard | Is the reference standard likely to correctly classify the target condition? | YES ☺ | A composite reference standard including multiple cultures and 8 week follow-up was used |
|  | Were the reference standard results interpreted without knowledge of the results of the index test? | YES ☺ | Antibody responses (=index test results) were not visible to the laboratory technicians (the MBio instrument did not display results) |
| Flow and Timing | Was there an appropriate interval between index test(s) and reference standard? | YES ☺ | Initial sputum samples and blood samples were collected on the same day. Standard 8 week follow-up was used. |
|  | Did all patients receive a reference standard? | YES ☺ |  |
|  | Did all patients receive the same reference standard? | YES ☺ | Patients with missing reference standard data were excluded from the analysis. |
|  | Were all patients included in the analysis? | NO ☹ | The number of patients enrolled differs from the number of patients included in the analysis due to the exclusion of 89 of 844 patients. Reasons for exclusion are described in the flow diagram. |


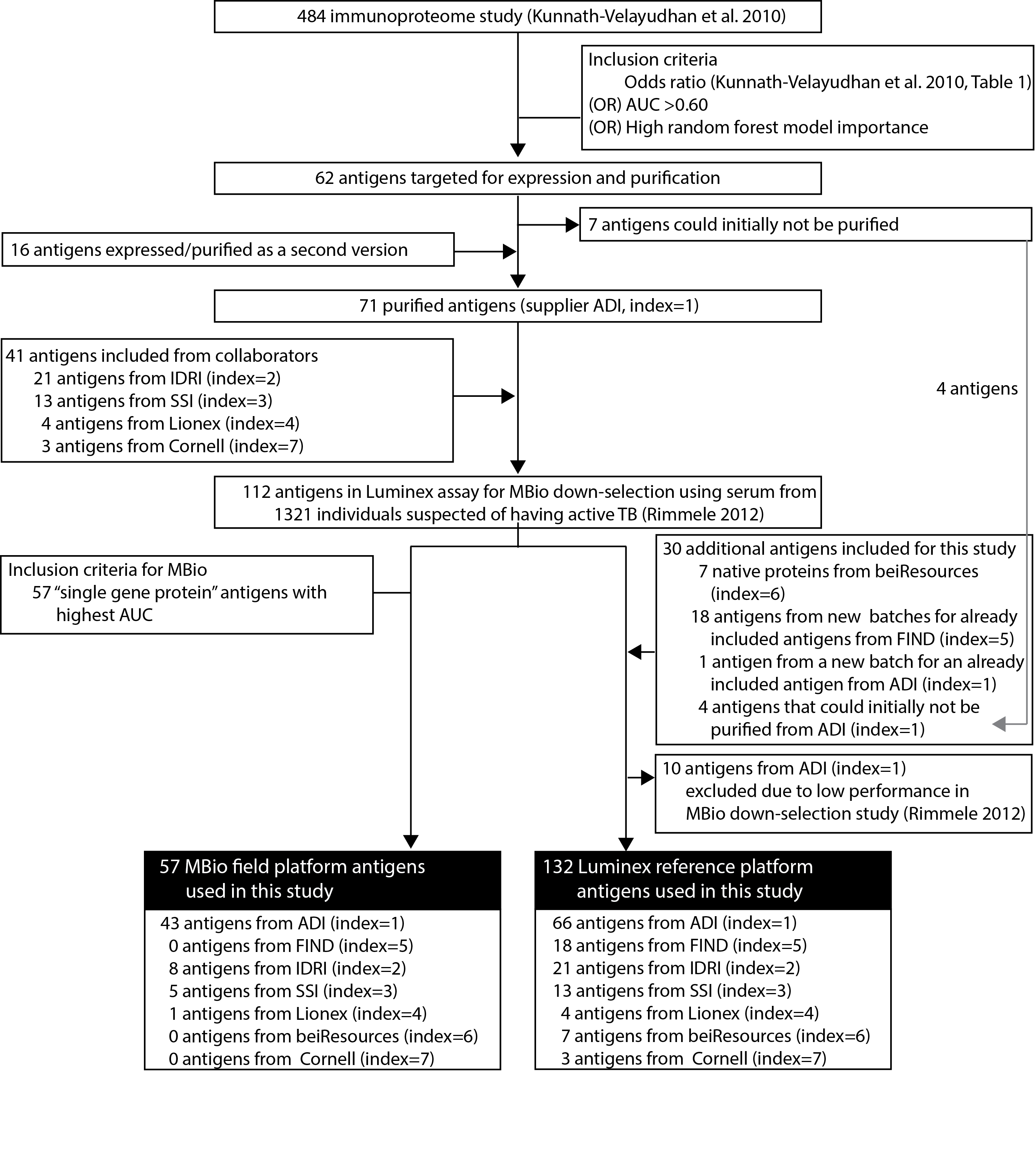


Figure S1: Antigen selection


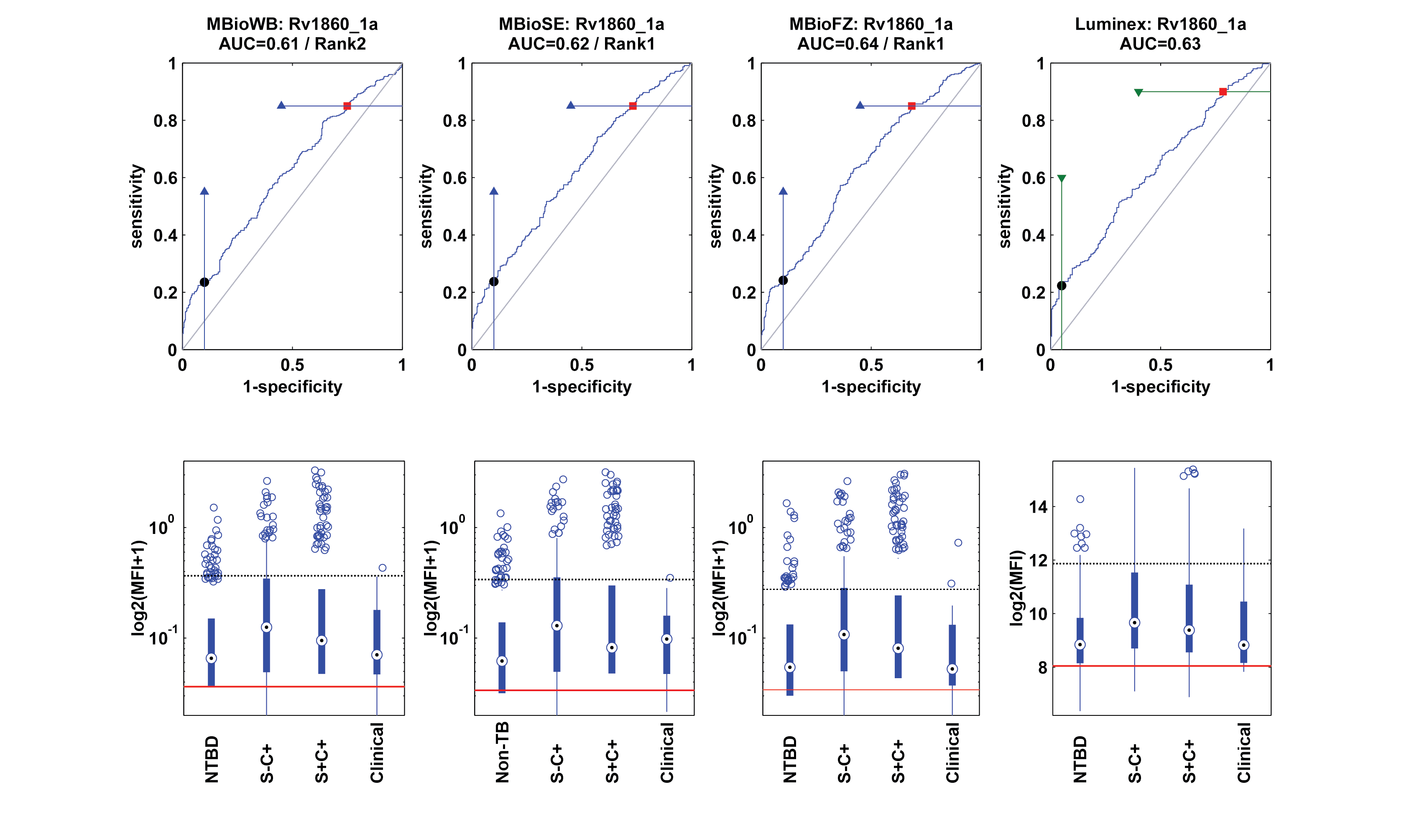


Figure S2: ROC curves (top) and boxplots (bottom) for Rv1860_1a that reached the best single antigen performance on MBio. Performance targets for both a TB detection test and TB triage test (
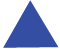
 mark field performance target,
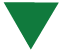
 mark reference performance targets) were not met. Cut-offs are based on pre-set specificity for the TB detection test (marked as
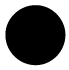
 in ROC curves and black dotted lines in boxplots) and on pre-set sensitivity for TB triage test (marked as
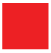
 in ROC curves and red solid lines in boxplots). (SE=fresh serum, WB=fresh whole blood, FZ=frozen serum, S=smear, C=culture, NTBD=Non-TB Disease)
